# Involvement of the Endothelin Receptor Type A in the Cardiovascular Inflammatory Response Following Scorpion Envenomation

**DOI:** 10.3390/toxins12060389

**Published:** 2020-06-12

**Authors:** Amina Sifi, Sonia Adi-Bessalem, Fatima Laraba-Djebari

**Affiliations:** Laboratory of Cellular and Molecular Biology, Faculty of Biological Sciences, USTHB, BP 32 El-Alia, Bab Ezzouar, Algiers 16111, Algeria;; aminasifi04@gmail.com (A.S.); flaraba@hotmail.com (F.L.-D.)

**Keywords:** scorpion envenomation, cardiovascular system, endothelin-1, ETA-R, inflammation, oxidative stress, matrix metalloproteinases (MMP-2 and MMP-9)

## Abstract

Elevated levels of endothelin-1 (ET-1) were recorded in sera of scorpion sting patients. However, no studies focused on the mechanism of ET-1 involvement in the pathogenesis of scorpion envenomation, particularly in the cardiovascular system which is seriously affected in severe cases of scorpion stings. Inflammation induced by *Androctonus australis hector* (*Aah*) scorpion venom in the heart together with the aorta was studied in mice pretreated with a specific endothelin A receptor (ETA-R) inhibitor. ETA-R inhibition resulted in the attenuation of the high amounts of cytokine (tumor necrosis factor alpha (TNF-α) and interleukin-17 (IL-17)) recorded in the sera of envenomed mice. The recovery of the oxidative stress marker balance and matrix metalloproteinase (MMP) expression were also observed, concomitantly with the reduction of tissular neutrophil infiltration. Additionally, the cardiac and the aortic tissue alterations, and the metabolic enzymes (creatine kinase (CK) and muscle–brain isoform creatine kinase (CK-MB)) overspread into sera were significantly attenuated. Obtained results suggest the implication of endothelin throughout its ETA receptors in the inflammatory response observed in the cardiovascular components during scorpion envenomation. Further knowledge is needed to better understand the implication of the endothelin axis and to improve the therapeutic management of severe scorpion sting cases.

## 1. Introduction

Scorpion sting envenomation is, in the majority of severe cases, the cause of heart failure and hemodynamic changes that can lead to pulmonary edema [1,2,3,4,5,6,7,8,9]. The etiology of the cardiovascular manifestations observed after scorpion envenomation is due to venom effects on the sympathetic nervous system and/or on the myocardium itself, as well as to the release of cathecholamines, neuropeptide Y(NPY), angiotensin II, and endothelin-1 (ET-1) induced after envenomation [6,10,11,12,13,14,15,16]. Proteases such as the angiotensin-converting enzyme (ACE) and endothelin-converting enzyme (ECE), isolated from *Tityus* species venom, may also contribute to the cardiovascular perturbations, including hemodynamic disorders, observed in human envenomation victims [17,18]. The inflammatory process and the overproduction of free radicals are frequently triggered by scorpion venom and promote cardiac injury [7,16,19,20]. Generally, venom components bind to the pattern recognition receptors (PRRs) of the innate immune system (Toll-like receptors, TLR2 and TLR4), activate nuclear factor kappa-B (NF-κB) transcription factor, and increase pro-inflammatory mediators and oxygen free radicals (ROS), which then leads to cardiac oxidative stress and cardiotoxicity [7,21,22,23,24,25,26].

Thus, the origin of the disturbances caused by scorpion venoms in the cardiovascular system may be multifactorial, involving several biological mechanisms [16,19,20,27]. According to some studies, elevated circulating concentrations of the effective vasoconstrictor endothelin-1 (ET-1) in the sera of envenomed victims and experimental models were associated with cardiovascular perturbations related to the hemodynamic disorders affecting blood pressure, cardiac rhythm, and heart rate [10,28,29]. ET-1 via the activation of its endothelin A receptor (ETA-R) triggers and/or modulates inflammatory reactions, causing vascular cells fibrosis and the production of reactive oxygen species [30,31,32,33,34]. Moreover, ET-1 can increase the production of superoxide anion and proinflammatory cytokines, including interleukin-1 (IL-1), tumor necrosis factor alpha (TNF-α), and IL-6 [30,31,33,34]. However, its role in these processes remains somewhat unclear and is not sufficiently studied, especially in the case of scorpion envenomation.

The objective of this study is to investigate the involvement of the endogenous endothelin type 1 in the inflammation response caused by *Androctonus australis hector* (*Aah*) scorpion venom in cardiac and aortic tissues of mice pretreated with the selective ETA-R antagonist, BQ123, and then envenomed.

## 2. Results

### 2.1. ETA Receptors Involvement in Venom-Induced Cytokines

**Figure 1:**
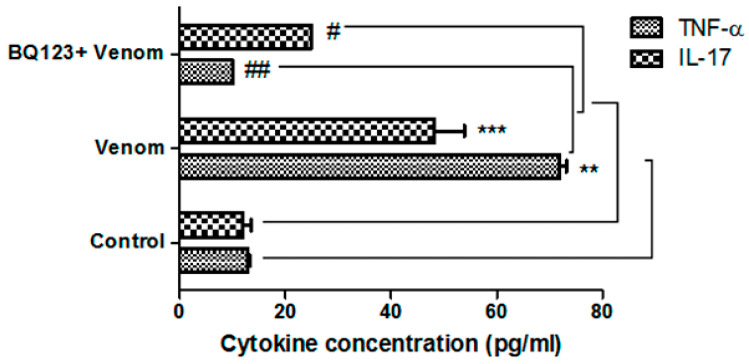
*Androctonus australis hector* (*Aah*) venom induced tumor necrosis factor alpha (TNF-α) and interleukin-17 (IL-17) production in sera in the presence or absence of the endothelin A (ETA) receptor inhibitor. Data are presented as the mean ± standard error of the mean (SEM) (*n* = 3). ** *p* ˂ 0.01, *** *p* ˂ 0.001 compared with control; ^#^ *p* ˂ 0.05, ^##^ *p* ˂ 0.01 compared with envenomed animals.

The release of TNF-α and IL-17, pro-inflammatory cytokines and mediators of cardiovascular diseases [35,36], was evaluated after experimental envenomation with *Aah* venom in the presence or absence of the selective ETA-R antagonist, BQ123. Results show a significant increase in both TNF-α (*p* < 0.01) and IL-17 (*p* < 0.001) levels after the injection of venom compared with the control mice (Figure 1). The inhibition of the ETA receptor prior to the injection of the venom led to a significant decrease in TNF-α (*p* < 0.01) and IL-17 (*p* < 0.05) levels compared to the envenomed animals (Figure 1). These data indicated that ETA could modulate TNF-α and IL-17 expression in envenomed models with *Aah* venom.

### 2.2. Role of ETA Receptors in Neutrophil Tissue Infiltration and Metalloproteinase Expression

**Figure 2:**
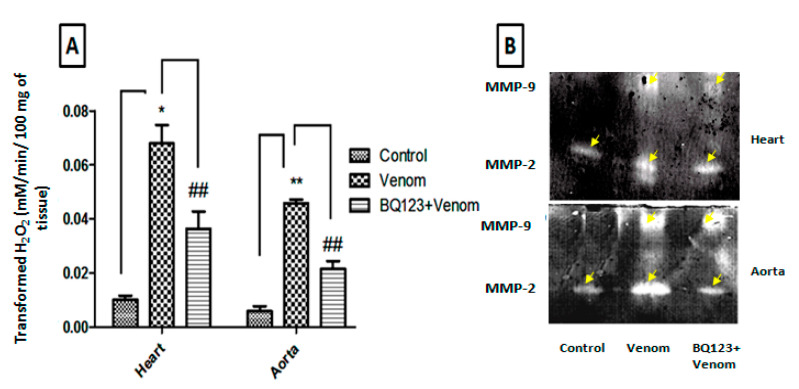
*Aah* venom induced neutrophil infiltration and gelatinase overexpression in the presence or absence of the ETA receptor antagonist. (**A**) Activity of myeloperoxidase (MPO) in the cardiac and aortic homogenates. (**B**) Expression of metalloproteinase-2 (MMP-2) and MMP-9 into cardiac and aortic tissue homogenates of mice. Data are presented as the mean ± SEM (*n* = 3). * *p* ˂ 0.05, ** *p* ˂ 0.01, compared with control group; ^##^ *p* ˂ 0.01 compared to envenomed animals.

Myeloperoxidase (MPO), the most abundant component of azurophilic granules of leukocytes, is secreted following neutrophil activation, contributing to innate host defenses [36,37]. The evaluation of MPO enzymatic activity in the cardiac (*p* < 0.05) and the aortic (*p* < 0.01) homogenates revealed a significant elevation in the studied tissues of the envenomed animals when compared to the controls (Figure 2A). The pretreatment with BQ123 prior to venom injection decreased the activity of MPO (*p* < 0.01) as compared to the envenomed group (Figure 2A). Thus, it seems that ETA receptors are involved in the activation and/or sequestration of neutrophils into the tissues.

Metalloproteinases (MMPs), are metal ion-dependent enzymes, and they are involved in matrix degradation, migration of cells, and tissue remodeling [38]. MMPs are associated with cardiovascular diseases, in particular MMP-2 and MMP-9 [39]. To evaluate the effects of venom on the secretion of MMP-9 and MMP-2, as well as the implication of the ETA receptor in this process, a gelatin zymography was performed.

Zymograms revealed increased activities of MMP-2 and MMP-9 in tissue homogenates of envenomed animals compared to the control samples (Figure 2B). However, the use of BQ123 seemed to be efficient against the expression of MMPs in the heart and the aorta in comparison with the envenomed animals (Figure 2B). These data indicated the ability of the scorpion venom to activate MMP expression and the involvement of the ETA receptor in this process.

### 2.3. ETA Receptor’s Role in the Oxidative Imbalance Caused by Aah Venom

**Figure 3:**
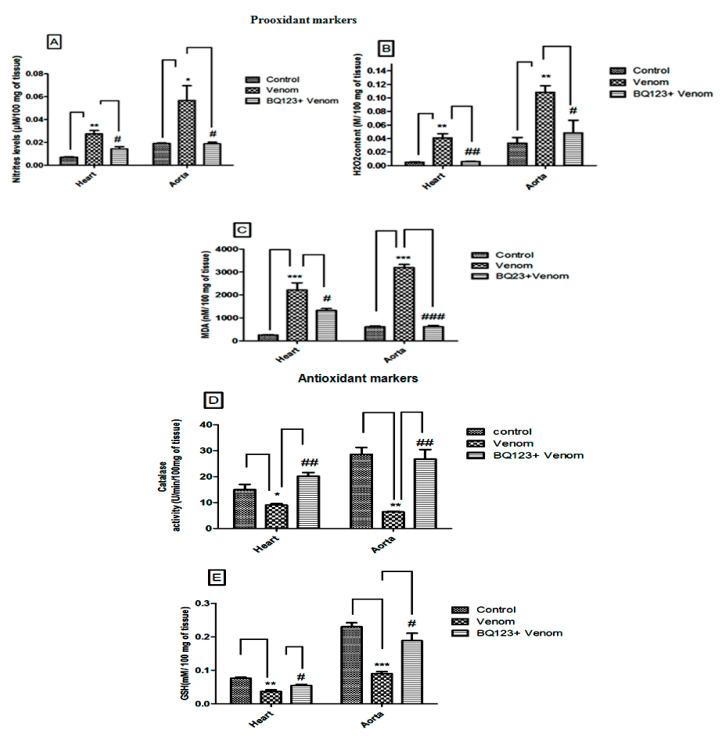
*Aah* venom induced oxidative stress imbalance with or without BQ123, the ETA receptor antagonist pretreatment. (**A**) Nitrite levels, (**B**) H_2_O_2_ amounts, (**C**) MDA levels, (**D**) level of glutathione (GSH), and (**E**) activity of catalase in homogenates of the heart and the aorta. Values are represented as the mean ± SEM (*n* = 3). * *p* ˂ 0.05, ** *p* ˂ 0.01, *** *p* ˂ 0.001 compared with controls; ^#^ *p* ˂ 0.05, ^##^ *p* ˂ 0.01, ^###^ *p* ˂ 0.001 compared with envenomed animals.

The evaluation of the production of nitric oxide, hydrogen peroxide, and lipid peroxidation in the cardiac and the aortic supernatants was performed by measuring nitrite (NO_2_^−^), hydrogen peroxide (H_2_O_2_), and malondialdehyde (MDA) after 24 h of envenomation. Results show significantly high levels of NO_2_^−^ (*p* < 0.01, *p* < 0.05), MDA (*p* < 0.001, *p* < 0.001), and H_2_O_2_ (*p* < 0.01, *p* < 0.01) in the heart and the aorta of envenomed mice when compared to their controls (Figure 3A–C, respectively). These results were accompanied by a significant reduction of the antioxidant parameters catalase (CAT) and glutathione (GSH) in both the heart (*p* < 0.05, *p* < 0.01) and the aorta (*p* < 0.01, *p* < 0.001) (Figure 3D,E).

Oxidative stress imbalance induced by *Aah* venom in the cardiac and aortic homogenates of experimental models was previously reported [16,19,20,23].

The inhibition of ETA receptors revealed a normalization of NO_2_^−^ (*p* < 0.05), MDA (*p* < 0.05), and H_2_O_2_ (*p* < 0.01) evaluated in the cardiac supernatants (Figure 3A–C, respectively). In the aorta, antagonism of the ETA receptors appears to prevent the excessive release of venom-induced reactive oxygen and nitrogen (NO_2_^−^ *p* < 0.05, MDA *p* < 0.001, and H_2_O_2_ *p* < 0.05) species (Figure 3A–C, respectively). The antioxidant parameters, catalase (CAT) and glutathione (GSH), were also stabilized in the cardiac (*p* < 0.01, *p* < 0.05) and the aortic *(p* < 0.01, *p* < 0.05) tissues (Figure 3D,E, respectively).

These data suggest the implication of the ETA receptor in the oxidative stress imbalance induced by scorpion venom.

### 2.4. Implication of ETA Receptors in Tissue Alterations and Metabolic Perturbations Induced by the Venom

**Figure 4:**
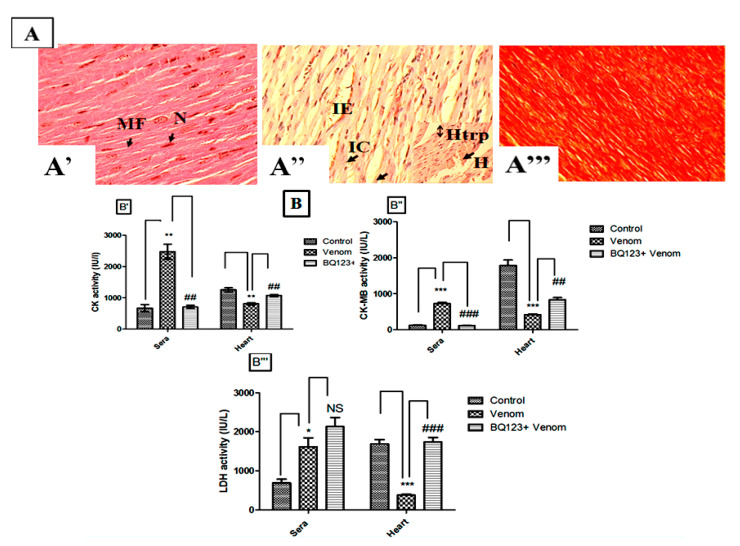
Evaluation of the cardiac function. (**A**) Histological analysis of the myocardium (A’: control, A’’: envenomed or A’’’: pretreated of envenomed mice with the ETA receptor antagonist). H: hemorrhage, Htrph: hypertrophy, IC: immune cell, IE: interstitial edema, MF: myocardial fiber, N: nuclei. (**B**) Enzyme activities of some cardiac markers in the sera and the cardiac homogenates (B’ control, B’’ envenomed mice, B’’’ mice pretreated with the ETA receptor antagonist). Data are presented as the mean ± SEM (*n* = 3). * *p* ˂ 0.05, ** *p* ˂ 0.01, *** *p* ˂ 0.001 compared with control group; ^##^ *p* ˂ 0.01, ^###^ *p* ˂ 0.001 compared with envenomed animals, NS: non-significant.

Microscopic tissue analysis revealed severe myocardial damage, muscle fiber degeneration, hemorrhagic areas, and edema. Significant myonecrosis with inflammatory cells was also observed in the envenomed animals compared to the controls where a normal architecture of the myocardium was observed (Figure 4A). The pretreatment with BQ123 seems to decrease myonecrosis, inflammatory cell infiltration, and edema induced by the venom (Figure 4A).

Specific markers of cardiac lesions, creatine kinase (CK), creatine kinase myocardial band (CK-MB) isoenzyme, and lactate dehydrogenase (LDH) activity were recorded to confirm the myocardial injury following venom injection. Results reveal that serum enzyme activities of envenomed mice were significantly increased against a reduction in the myocardium tissue compared to the control values (Figure 4B). The pretreatment with BQ123 before venom injection significantly restored the activities of CK (*p* < 0.01), CK-MB isoenzyme (*p* < 0.01), and LDH (*p* < 0.001) in the myocardium (Figure 4B). These results indicate a cardioprotection exerted by BQ-123 by preserving the cellular membrane integrity and restricting leakage of CK and CK-MB isoenzyme and LDH through the membranes.

**Figure 5:**
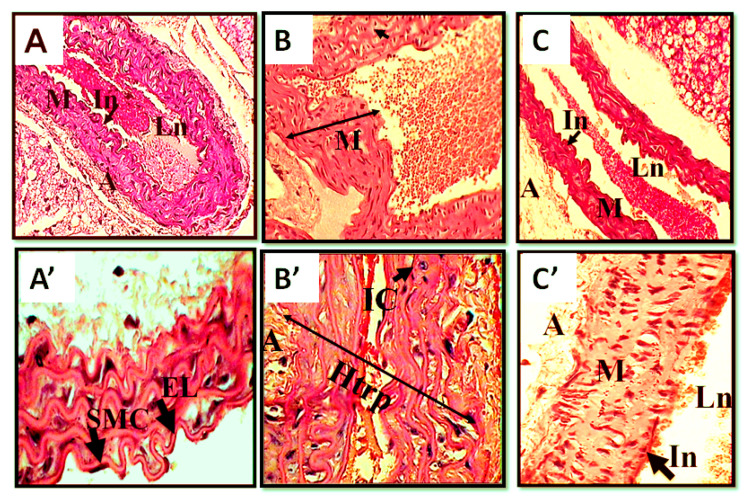
Histological sections of aorta of control and envenomed animals, in the presence and absence of BQ123, 24 h after envenoming. Thin sections of 5 μm. (**A**) Control. (**B**) Aortic tissue of mice injected with scorpion venom. (**C**) Aortic tissue of envenomed mice pretreated with BQ123, the ETA receptor antagonist. A: adventice, EL: elastic lamellae, Htrp: hypertrophy, IC: immune cell, In: intima; Ln: lumen M: media; SMC: smooth muscle cells. Hematoxylin–eosin stain, A, B, C magnification 400×. A′, B′, C′ magnification 1000×.

The histological sections of the aorta, as illustrated in Figure 5, showed an important aneurism of the artery and hypertrophy of the media caused by the venom, when compared with controls. An inflammatory cell infiltration was also observed (Figure 5B, B’). In contrast, inhibition of the ETA-R revealed a reduction of the structural disorganization induced by the venom and the extent of dilation of the aortic artery (Figure 5C, C’).

## 3. Discussion

Elevation of ET-1 levels in the plasma of stung victims was reported in some scorpion envenomation [10,21,28,29]. However, no studies focused on its role in the inflammatory response induced by scorpion venom. In this study, the pathogenic role of ET-1 through its ETA-R in the development of the cardiovascular dysfunction induced in envenomed mice is discussed.

Our results demonstrate that the administration of *Aah* venom to mice seems to cause an important increase in sera levels of TNF-α and IL-17. TNF-α is a potent proinflammatory cytokine involved in cardiac injuries, promoting inflammation, apoptosis, and accumulation of extracellular matrix (ECM) [40,41]. IL-17 could also be implicated in cardiovascular diseases [35]. In early studies, it was reported the activation of the vascular endothelium by the expression of proinflammatory cytokines such as TNF-α and IL-1β, induced adhesion molecules (e.g., E-selectin), and chemokines (e.g., chemokine CXC motif ligand-8 (CXCL8)) that play a pivotal role in the cascade of leukocyte migration into the sites of inflammation [42,43,44]. Moreover, *in vitro* and *in vivo* studies revealed that IL-17 contributes to the inflammation by modulating endothelial activation and neutrophil recruitment [44,45]. Furthermore, IL-17 can upregulate and/or function synergistically with local mediators of inflammation including TNF-α, and it enhances the ECM injury through the activation of matrix metalloproteinases (MMPs) production and the inhibition of matrix repair component synthesis, such as proteoglycans and collagens [46]. This would partially explain MMP-2 and MMP-9 gelatinase overexpression in tissue homogenates of envenomed mice. MMPs are particularly involved in the degradation of the ECM to facilitate the progression of cell migration [47], including neutrophils. This explains the elevated activity of MPO in the tissues of the envenomed animals. Neutrophils are one of the most important sources of reactive oxygen intermediates, namely H_2_O_2_ and NO [19], whose high levels were observed in tissues after envenomation. In addition to H_2_O_2_ and nitrites, an increase in lipid peroxidation in the cardiac and the aortic homogenates was observed. Lipid peroxidation causes protein damage and activates enzymes, either via a direct attack by free radicals or via chemical modification of its final products [48]. Early studies reported the generation of NO and lipid peroxidation in envenomation by scorpions [19,23,49]. Reactive oxygen species and nitric oxide overproduction is generally accompanied by a severe imbalance of the redox status with altered or insufficient antioxidant defense [50], reflected in this study by the attenuation of catalase activity and glutathione content. The previously described effects of the venom were associated with tissue damage in the heart and the aorta and the release of the enzymatic cell contents, namely, LDH, CK, and CK-MB in sera, as already described in our previous study and others [2,3,7,9,16,19,51,52].

In this study, the inhibition of the ETA-R of endothelin by the administration of the specific antagonist BQ123 prior to the venom decreased TNF-α and IL-17 levels in sera. It was reported that ET-1 induces activation of macrophages leading to the release of pro-inflammatory and chemotactic mediators, including TNF-α [53,54]. Therefore, ET-1 can indirectly enhance the expression of adhesion molecules on stimulated vascular endothelial cells by TNF-α and promote the migration of the polymorphonuclear neutrophils [55,56]. IL-17 is also involved in the migration of neutrophils [57]. However, the role of ET-1 in IL-17 release or IL-17-induced cell migration in cardiovascular diseases remains poorly defined. It was reported that the blockade of ETA-R attenuates neutrophil accumulation and myeloperoxidase activity in the ischemic myocardium [58]. This could partially explain the attenuation of MPO activity in this study, following the inhibition of the ETA-R. The reduced activity of MPO in our model reflects the reduction of neutrophil infiltration, which could be associated with a reduction in the expression of MMPs. One possible explanation for ET-1 regulation of gelatinases could be via the mitogen-activated protein kinase (MAPK) signaling pathways, through the ETA-R [59,60]. Other factors whose expression is mediated or co-regulated through ET-1 receptor pathways are also implicated in the regulation of MMP expression, such as osteopontin or angiotensin II. At the same time, the signaling pathways downstream of the ETB receptor cannot be excluded and it is not excluded that enhanced endothelial ETB receptor activation following the selective ETA-R blockade may lead to the increase in ETB-mediated signaling [59].

The results of the present study also show that the blockade of the ETA-R resulted in the recovery of the oxidative balance. This could be partially explained by the attenuation of leukocyte infiltration into injuries, as they are the major source of reactive oxygen species [61,62]. At the same time, multiple researches support the role of ET-1 in ROS generation and endothelial dysfunction. ET-1 stimulates ROS generation in human endothelial and vascular smooth muscle cell cultures, in addition to isolated vessels [63,64,65]. It was earlier reported that the blockade of the endothelin system by using selective receptor blockers, including BQ123, was efficient in reducing lipid peroxidation, while it increased the activities of antioxidant enzymes such as superoxide dismutase (SOD) and catalase and the concentration of total glutathione [66,67,68,69]. Our results are in accordance with these observations and with a previous report which demonstrated that ET-1 induced oxidative stress by decreasing glutathione, reducing the antioxidant GSH/glutathione disulfide (GSSG) ratio and inducing lipid peroxidation in a time-dependent manner [70]. ROS generation leads to the activation of NF-ĸB which, in turn, stimulates the synthesis of pro-inflammatory cytokines, chemokines, and adhesion molecules, associated with the development of the inflammatory response [71]. In this context, it was demonstrated that ET-1 stimulates the peripheral blood mononuclear cells and tissular cells to release TNF- α. This increases the generation of ROS in various cell types via signaling with NF-*κ*B and nicotinamide adenine dinucleotide phosphate (NADPH)-oxidase [72,73,74]. Thus, inflammation and oxidative stress constitute a vicious cycle in the development of endothelial dysfunction, which is carried out with the active participation of ET-1. The present results are confirmed by the prevention of the severe histological alterations and enzymes release induced by the venom, in the heart and the aorta tissue of mice.

## 4. Conclusions

In conclusion, this study provides evidences that the endothelin axis, through the ETA-R of ET-1, is implicated in the inflammatory response engendered by the venom in the cardiovascular system. Thus, the result of *Aah* scorpion venom and its components on the vascular endothelium would lead to its alteration, causing the release of ET-1. This, through the ETA-R, induces the vasoconstriction and then the alteration of the coronary endothelium, provoking a diffuse myocardial ischemia. ET-1 through its ETA-R, which is also expressed in endothelial cells, smooth muscle cells, and cardiomyocytes, would lead to the damage of the cardiac tissue and an aortic aneurysm by the activation of various proinflammatory pathways, mainly that of NADPH oxidase, but also the activation and the recruitment of inflammatory cells including macrophages and polymorphonuclear neutrophils (PMN) with the release of proinflammatory mediators and MMP overexpression, thereby resulting in an overall cardiovascular dysfunction. However, other studies are required to examine more closely the molecular mechanism that controls the endothelin axis in the pathogenesis of scorpion envenomation.

## 5. Materials and Methods

### 5.1. Venom

Lyophilized venom of *Androctonus australis hector* (*Aah*) scorpion was obtained from the Laboratory of Cellular and Molecular Biology of the Biological Sciences Faculty at USTHB (Algiers, Algeria).

### 5.2. Animals

A total of 24 N.M.R.I male mice (20 ± 2 g body weight) were used in this study. They were purchased from the animal breeding of Pasteur Institute of Algeria. The animals had ad libitum access to water and food. All the experiments on mice were carried out in accordance with guidelines for the care of laboratory animals and approved by the European Community Council Directive (86/609/EEC). This study was approved then by the Deontology and Ethic Committee of the Research Thematic Agency in Health Science (ATRSS) formerly National Agency of Research Development in Health (ANDRS). Its number code is N°42-ANDRS-2011. The project approval was then obtained by the National Committee for the Evaluation and Programming of University Research funded by the Algerian Ministry of Higher Education and Scientific Research on 1 January 2016 (Code: D01N01UN160420150005).

### 5.3. Chemical Reagents and Drugs 

The used chemicals and reagents were mostly from Biochem (Montréal, Canada), Merck (Darmstadt, Germany), Prolabo (Darmstadt, Germany), and Sigma Aldrich (St. Louis, MO, USA). All the reagents were of analytical grade.

The drug used in this study, BQ123 (Sigma Aldrich), was dissolved in a saline solution (NaCl 0.9% (*w*/*v*).

### 5.4. Experimental Design

Mice were separated into three batches of eight animals each. The first batch was the control and received a vehicle (NaCl 0.9%, *w*/*v*; subcutaneous route). The second batch received a subcutaneous injection of a sublethal dose of *Aah* venom (0.5 mg/kg), and the last group was pretreated with the ETA receptor antagonist (BQ123; 0.5 mg/kg; 30 min before the venom; intravenous route). Animals were euthanized in the 24 h following the injection of saline or venom. Blood was collected for the measurement of some metabolic parameters. The heart and the aorta were weighed, homogenized (1:10, *w*/*v*) in a physiological saline solution (NaCl 0.9%, *w*/*v*) with a polytron homogenizer (T10 Ultra-turax), and centrifuged at 2486× *g* for 20 min. Supernatants with pellets were used for the different assays.

### 5.5. Cytokine Level Measurement

TNF-α and IL-17 cytokines were assayed in sera by a colorimetric two-site sandwich ELISA using a specific mouse-kit (Sigma, St. Louis, MO, USA). Assays were performed according to the manufacturers’ instructions, and the reaction product had an absorbance maximum at 450 nm. Concentrations were determined from standard curves and expressed as pg of cytokine per mL of serum. The detection sensitivity was 3 pg per mL for the TNF-α, and the minimum detectable dose of IL-17 is typically less than 6 pg per mL.

### 5.6. Evaluation of the Myeloperoxidase Activity

The accumulation and sequestration of neutrophils into the studied tissues was carried out by assaying the myeloperoxidase (MPO) activity. MPO activity was determined as described by Krawisz [75]. Tissue homogenates were added to the substrate, *O*-dianisidine (0.167 mg/mL), dissolved in phosphate buffer 0.05 M; pH 6 and H_2_O_2_ (0.4 mM). The absorbance was read at 460 nm, and results are indicated as transformed H_2_O_2_ mM per min per 100 mg of tissue, using a molar extinction coefficient of 11.3 mM^−1^∙cm^−1^.

### 5.7. Evaluation of Oxidative Stress Biomarkers in Tissues

#### 5.7.1. Determination of Prooxidant Contents and Lipid Peroxidation

Prooxidant contents were evaluated by the measurement of nitrites and hydrogen peroxide amounts and lipid peroxidation.

Nitrite (NO_2_^−^) levels were measured using Griess reagent according to the method described by Sun [76]. Samples were deproteinized with trichloroacetic acid at 10% (*v*/*v*) for 1 h at 4 °C followed by a centrifugation at 1466× *g* for 10 min. The resulting supernatants were incubated with Griess reagent (*v*/*v*) for 20 min at room temperature. Absorbance was read at 540 nm, and concentrations expressed in µM per 100 mg of tissue were calculated by the extrapolation of the optical density (OD) standard curve previously established from an NaNO_2_ solution.

Hydrogen peroxide (H_2_O_2_) was also measured, based on phenol red oxidation by horseradish peroxidase (HRP), following Pick and Keisari’s method [77]. The metabolite was assayed in the cardiac and aortic supernatants that were brought into contact with a solution of phenol red containing 5.5 mM glucose, 0.28 M of phenol red, and 8.5 U/mL of HRP in phosphate buffered saline (PBS) buffer, pH 7, in a 96-well microplate. After 1 h of incubation at 37 °C, 10 µM of NaOH (1 N) was added in order to stop the reaction, and the absorbance was read at 620 nm. H_2_O_2_ concentrations were calculated from a standard curve prepared with concentrations from 5 nM to 10 µM of H_2_O_2_.

Lipid peroxidation was evaluated by the measurement of MDA content. MDA was quantified in the supernatants following Ohkawa’s method using thiobarbituric acid (TBA). The interaction between MDA and two molecules of TBA allows the formation in acid of a warm medium with pink pigment whose optical density is measured at 532 nm [78]. Results are expressed as nM of malondialdehyde formed by 100 mg of tissue.

#### 5.7.2. Determination of Antioxidant Contents

Antioxidant markers, catalase activity, and glutathione levels were assayed. The evaluation of catalase enzyme activity was carried out using the method of Aebi, based on the degradation of hydrogen peroxide (H_2_O_2_) in water and oxygen under the action of catalase. The absorbance was measured at 240 nm every 30 s for 3 min [79]. The enzymatic activity is expressed in units of catalase per 100 mg of tissue.

Glutathione **(**GSH) amounts were determined following Ellman’s procedure based on the interaction of DTNB (5,5-dithiobis2-nitrobenzoic acid) with samples that contained sulfhydryl groups at 412 nm [80]. Total GSH content was indicated as mM GSH per 100 mg of tissue.

### 5.8. Gelatin Zymography 

According to the method of Hu [81], gelatin zymograms were performed on 8% SDS-PAGE gels, containing gelatin at 1.5 mg per mL. Extracts of the cardiac and aortic tissues (20 μg of protein) were prepared in non-reducing conditions. Electrophoresis was carried out at room temperature using 120 V. After the run, gels were washed twice for 20 min in 2.5% Triton X-100 to remove SDS, then incubated in Tris-HCl buffer (50 mM, pH7.4) containing NaCl (200 mM) and CaCl_2_ (5 mM) for 18 h at 37 °C. Gels were then stained with Coomassie blue R250 dye and destained with a solution composed of 50% methanol, 10% acetic acid, and 40% water. The presence of gelatin-degrading enzymes is indicated by the clear zones of substrate lysis against a blue background stain.

### 5.9. Enzyme Assays

Enzyme activities of lactate dehydrogenase (LDH), creatine-kinase (CK), and creatine-kinase M-B (CK-MB) were determined in tissues and sera, according to the manufacturers’ (Spinreact, Spain) instructions. The enzyme activity was assessed using a spectrophotometer set at 340 nm wavelength, and results are expressed in international units (IU/l).

### 5.10. Histological Section Analysis

The hearts and aorta were fixed in buffered formalin (10%) at room temperature during 48 h. They were embedded in paraffin, then sectioned with transversal slices of 5 µm submitted to a hematoxylin–eosin staining. The observation of the tissue sections was carried out with an optical microscope connected with a Motic Digital Microscope PAL System camera (Meyer Instruments, Houston, TX, USA).

### 5.11. Statistical Analysis of Data

Results are presented as means ± standard error of the mean (SEM). Variations between groups were analyzed by the Student *t*-test (Graph Pad Prism 5 Software, San Diego, CA, USA). Values of probability less than 5% (*p* ˂ 0.05) were considered significant.
